# Genetic variants of *Dabie bandavirus*: classification and biological/clinical implications

**DOI:** 10.1186/s12985-023-02033-y

**Published:** 2023-04-14

**Authors:** Bingyan Liu, Jie Zhu, Tengfei He, Zhenhua Zhang

**Affiliations:** Institute of Clinical Virology, Department of Infectious Diseases, The Second Affiliated Hospital of Anhui Medical University, Furong Road 678, Hefei, 230601 China

**Keywords:** *Dabie bandavirus* (DBV), Severe fever with thrombocytopenia syndrome (SFTS), Genotype, Evolutionary rate, Distribution of DBV, Genetic variation

## Abstract

Severe fever with thrombocytopenia syndrome (SFTS) is an emerging infectious disease caused by *Dabie bandavirus* (DBV), a novel Bandavirus in the family Phenuiviridae. The first case of SFTS was reported in China, followed by cases in Japan, South Korea, Taiwan and Vietnam. With clinical manifestations including fever, leukopenia, thrombocytopenia, and gastrointestinal symptoms, SFTS has a fatality rate of approximately 10%. In recent years, an increasing number of viral strains have been isolated and sequenced, and several research groups have attempted to classify the different genotypes of DBV. Additionally, accumulating evidence indicates certain correlations between the genetic makeup and biological/clinical manifestations of the virus. Here, we attempted to evaluate the genetic classification of different groups, align the genotypic nomenclature in different studies, summarize the distribution of different genotypes, and review the biological and clinical implications of DBV genetic variations.

## Background

In 2009, Hubei and Henan provinces in China reported an emerging infectious disease characterized by severe fever with thrombocytopenia syndrome (SFTS). The major clinical symptoms of the disease were fever, myalgia, gastrointestinal symptoms, dizziness, arthralgia, chills, and local lymph node enlargement. Abnormal laboratory findings included thrombocytopenia, leukopenia, and elevated serum liver enzyme levels. Researchers isolated a new bunyavirus from serum specimens of patients in the acute phase and obtained its full-length sequence. The new bunyavirus was named *Dabie bandavirus*, also known as severe fever with thrombocytopenia syndrome virus (SFTSV), Huaiyang Shan virus (HYSV), or new bunyavirus (NBV) [1, 2]. According to the classification standards of the International Committee on Taxonomy of Viruses (ICTV, 2021), DBV belong to genus *Bandavirus* of the family *Phenuiviridae*. DBV infection is mainly transmitted through the bite of infected ticks, but can also be transmitted through direct contact with the bodily fluids of infected patients [3]. The incidence of SFTS is high among people over 40 years of age, and advanced age is a risk factor associated with SFTS severity and mortality [4]. In the last 10 years, SFTS has become endemic primarily in China, Japan, and South Korea and has occasionally occurred in other countries or regions, such as Vietnam. The reported death rates vary from 6 to 30%, but no effective vaccine or antiviral drug therapy is currently available against *Dabie bandavirus* [5–8].

DBV is a negative-strand RNA virus with three genomic segments: large (L), medium (M), and small (S) [9]. A variety of genotyping methods and nomenclatures have been developed for DBV, with most methods based on whole-genome sequencing and phylogenetic analysis. Genotypes are usually named based on the geographical distribution of the viral strains or phylogenetic analysis. Uppercase letters and numbers are used to denote different genotypes. However, no universal standard or nomenclature for genotyping this virus currently exist, and the results from different genotyping methods cannot be aligned. Statistical analysis has suggested correlations between genotypes and regions in the world and subregions within the same country, such as different provinces in China. Data have also suggested differences in lethality for populations and experimental animals among different genotypes [10].

Reassortment, recombination, and gene variants are important factors that affect the genetic diversity and molecular evolution of DBV. Similar to other RNA viruses, DBV lacks a proofreading mechanism during viral replication and transcription and exhibits high mutation rates [11, 12]. To date, studies exploring mutations of biological significance are scarce, with only a few focusing on RNA-dependent RNA polymerases (RdRps) (L segment) and Gn/Gc proteins (M segment). It is our strong belief that a systematic investigation of DBV variations can help reveal the molecular mechanisms of virus infection and provide insight for the development of therapies and vaccines against SFTS infection.

In this article, the evolutionary rate and genotyping methods for DBV and some variants that may affect the biological characteristics of the virus suggested by several studies were reviewed. The relationships between three genotyping methods and differences in virulence are also discussed.

## Structural features of the *Dabie bandavirus* genome

**Fig. 1:**
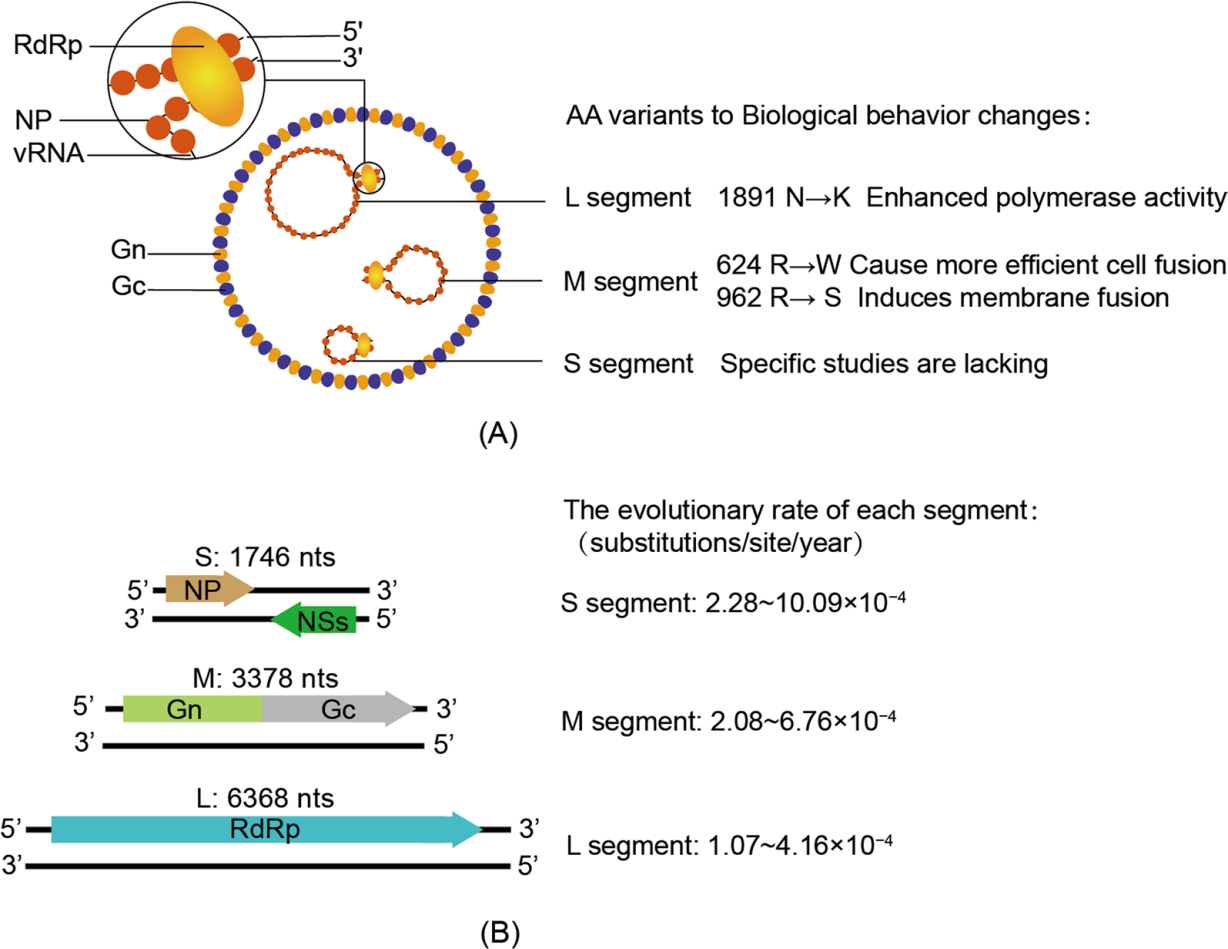
**A** DBV structure. Viral structure with its protein components and viral RNA, and the amino acid variation sites which alter the biological properties of DBV. **B** Diagram of the genomic structure of DBV and the evolutionary rate of each segment [42–46]

DBV is a novel virus belonging to the genus *Bandavirus* of the family *Phenuiviridae*. It is a spherical, membrane-enveloped virus with a diameter of 80–100 nm. The *Dabie bandavirus* genome, similar to that of other members of the order *Bunyavirales*, is composed of three negative-strand RNA segments: large (L), medium (M), and small (S) [9]. The genomic structure of DBV is shown in Fig. 1. Similar to that of other members of the genus *Phenuiviridae*, the terminal sequence of the DBV genome is highly conserved. Its 3ʹ and 5ʹ ends are complementary and form a panhandle structure, and each segment resembles a closed loop [1, 2]. The L fragment of DBV has a single open reading frame (ORF) of 6368 nucleotides, encoding an RdRp of 2084 amino acids, which is required for genome replication and transcription. The M segment consists of 3378 nucleotides with an ORF encoding a glycoprotein (GP) of 1073 amino acid precursors (Gn and Gc). Gn/Gc proteins recognize and bind receptors on the cell surface of the host and may bind to different cell surface receptors in various cell types. As shown by Hofmann et al., dendritic cell (DC)-specific intracellular adhesion grabbing non-integrin (DC-SIGN) binds to the Gc/Gn proteins of DBV and acts as an entry receptor for the virus. DC-SIGN is a C-type lectin mainly present on the surface of DCs [13, 14]. Non-muscle myosin heavy chain IIA, which is widely distributed in animal cells, can also facilitate DBV infection [15, 16]. The evidence above indicates a direct relationship between Gn/Gc proteins and DBV infection. The S segment is an ambisense single-stranded RNA molecule containing 1746 nucleotides and two ORFs oriented in opposite directions and spaced apart by 54 nucleotides. The ORF closer to the 5′end encodes the nonstructural protein (NSs), which is transcribed from the negative-sense templates. While nucleocapsid protein (NP) is encoded in a complementary RNA corresponding to the 3' half of the genomic S segment. NSs significantly represses type I IFN production by blocking cell signaling, thereby inhibiting the host antiviral immune response [17, 18]. NP has an important function in the protection of the viral genome, virus replication, and formation of inclusion bodies [19, 20]. The viral genome binds to the nucleocapsid to form a ribonucleoprotein (RNP) complex with the S, M, and L segments, and many RdRps attach to RNP [21–23].

## Single amino acid variations (SAVs) alter biological functions of DBV

### Amino acid substitutions at position 1891 of the L segment

The L segment of DBV has multiple functions, such as RNA polymerase activity, endonuclease activity, and other functions related to transcription and genome RNA replication [24]. The cap-snatching endonuclease in influenza viruses has been thoroughly characterized, and the cap-binding region is located in the third subunit of the PB2 domain, which corresponds to the C-terminal region of the bunyavirus L segment. B7, a subclone of the YG1 strain, has an amino acid substitution at position 1891 (N → K) in the L segment, and B7 shows strong plaque formation and cytopathic effects [25–27]. In IFA experiments, a greater amount of protein aggregation was observed in B7-infected cells, but the viral titers did not differ significantly among these sub-clones [28]. The cap-binding domain structure of Rift Valley fever phlebovirus (RVFV) and DBV is located in the C-terminal region of the L segment [24, 29]. The C-terminal domain of RNA polymerase not only affects viral replication but also affects pathogenicity and host range, as exemplified by the PB2 domain in influenza A virus [30, 31].

Noda et al. assessed the effects of a mutation at position 1891 on the activity of the L segment using a mini-replicon assay. The results suggested that a single amino acid substitution at position 1891 of the L segment did not affect its expression level or localization. Position 1891 is located near the boundary between the arm and lasso domains. The charge of the domain edges was affected when amino acid substitutions occurred at position 1891. Basic amino acids with a positive charge increased the activity of the viral polymerase, while glutamate with a negative charge reduced polymerase activity [28].

### Amino acid substitutions at position 624 in the Gc of the M segment

The three subclones E3, A4, and B7 of the YG1 strain displayed differences in fusion activity, and two amino acids were found to differ among the sequences of the envelope GPs [32]. Tsuda et al. investigated amino acid substitutions at positions 624(R → W) and 328(Y → H), demonstrating that the amino acid at position 624 played an important role in inducing cell fusion [33]. The GP with an amino acid substitution at position 624 induced more efficient cell fusion in transfected cells, without increasing the abundance of Gn and Gc proteins on the cell surface or within the cells. GP (Y624W) expression induced syncytium formation at a pH below 6.2. In addition, an amino acid substitution of tryptophan at position 624 enhanced cell fusion activity. Substitutions of glycine, serine, and aspartic acid achieved the same effect, but substitutions with arginine or lysine had no effect on cell fusion activity. Together, the study showed that fusion activity enhancement was not caused by surface expression of Gn but by sensitivity to acidic conditions [34].

Structural analysis of DBV and other viruses belonging to genus Phlebovirus suggest that membrane fusion is triggered by structural rearrangement under acidic conditions [35, 36]. Serine and aspartate are located at position 624 in DBV and the corresponding site in Uukuniemi virus (UUKV), respectively, and the two viruses induce syncytium formation below pH 6.2 and 5.8, respectively [37, 38]. The pH range of cell fusion in RVFV and UUKV was the same as that of SFSTV GP (Y624S) and GP (Y624D), demonstrating that the amino acid at position 624 plays an important role in the sensitivity of GPs to low-pH conditions for viruses in the genus Phlebovirus.

### Amino acid substitutions at position 962 in the Gc of the M segment

Tani et al. observed syncytium formation in cells expressing GPs from the YG-1 and SPL030 strains. However, in cells expressing GPs from the HB29 strain, syncytium formation was hardly induced regardless of pH. An amino acid substitution at position 962 of the GPs of HB29 (arginine → serine) (R962S) induced membrane fusion, whereas an amino acid replacement (serine → arginine) (S962R) in the GP of YG1 did not induce membrane fusion [39].

Arginine at position 624 in domain I and histidine at position 940 in domain III may affect viral infectivity through interaction with the fusion loop region [33, 36]. The serine at position 962, which is located in domain III, may interact or have strong interactions with this region. This may indicate that serine at position 962 in the GPs of DBV may be a significant factor in the induction of membrane fusion and viral infections, but further study is required to elucidate the underlying mechanisms [39].

### Other studies on amino acid substitutions

Brennan et al. found that an amino acid substitution at position 330 (F → S) of the M segment can lead to an increased number of larger foci in Vero cells, but the specific mechanism remains unknown [40]. Yun et al. found a unique amino acid substitution in the KAGWT strain at position 66 (A → S) of the Gn protein. Compared to other DBV strains, the Korean strains had 53 unique amino acid variations in the L, Gn, Gc, N, and NS proteins. However, the functional effects of these amino acid substitutions remain unclear [41].

## Evolutionary rate of DBV

Liu et al. calculated the evolutionary rate of each RNA segment of DBV using Bayesian analysis and found that the most recent common ancestor could be dated back approximately 182–294 years [42, 43]. Nevertheless, another study indicated that the average rate of evolution was 4.16 × 10^–4^ (s/s/y) for the L segment, 6.76 × 10^–4^ s/s/y for the M segment, and 10.9 × 10^–4^ s/s/y for the S segment. The rate of evolution was highest for the S segment: 1.61 times greater than the M segment and 2.62 times greater than the L segment [44]. However, these results differed significantly from those of other studies, suggesting that virus strains from different regions have different evolutionary rates, and that datasets consisting of different virus strains could bias the results (the evolutionary rates from each study are presented in Table 1) [10, 45, 46]. Together, these results suggest that DBV has a common ancestor but has evolved into different genotypes to adapt to environmental changes. Despite variations among individual studies, the L segment has repeatedly been found to be the most conserved segment in the viral genome of DBV. Freire et al. calculated the evolutionary rate of RVFV, a novel virus belonging to the genus *Phlebovirus* of the order *Bunyavirales*, finding it to be similar to that of DBV [47].

**Table 1:** Evolutionary rates from different studies (number of nucleotide substitutions/site/year × 10^−4^)

Virus name	Evolutionary rates		
*Bunyavirales*	S	M	L
Liu (DBV) [42]	2.28	2.42	1.19
Fu (DBV) [46]	5.07	2.84	1.87
Yun (DBV)[43]	2.60	2.08	1.07
Liu (DBV) [44]	10.9	6.76	4.16
Huang (DBV) [45]	3.17	3.09	1.96
Freire (RVFV) [47]	3.36	3.80	2.31
Chen (CCHFV) [49]	1.16	1.56	0.80
Chen (CCHFV without reassortants) [49]	6.55	1.45	1.69
*HBV*			
Osiowy (HBV) [50]	0.79		
*Influenza virus*			
Saitou (Influenza virus) [51]	41		

A study of Crimean-Congo hemorrhagic fever virus (CCHFV; order, *Bunyavirales*; family, *Nairoviridae*; genus, *Orthonairovirus*) showed that the existence of reassortants in the dataset could significantly affect the calculated evolutionary rate [48, 49]. As such, reassortants could lead to inconsistent results among studies. In this study, we compared the evolutionary rates of different viruses and found that the evolutionary rate of DBV, an RNA virus, was higher than that of DNA viruses [50]. Although DBV and influenza viruses are both members of negative-strand RNA viruses, the evolutionary rate of the influenza virus was much higher than that of DBV [51]. Nevertheless, even though evolutionary rates differed among different DBV studies, these differences were much smaller than the interspecific differences.

At the amino acid level, the ratio of the rates of non-synonymous to synonymous substitutions (dN/dS) in the M segment was 0.14. The dN/dS ratio for RdRp was 0.048. Using the dN/dS ratio, the selective pressure can be described. When amino acid changes lead to a selective disadvantage, the synonymous substitution rate will be higher than the nonsynonymous substitution rate (dN/dS < 1). Although values of dN/dS were low (less than 1) for all segments of DBV, this value was significantly higher for the M segment than the other segments [10]. This may be partially due to the fact that Gn/Gc proteins comprise the essential factor by which DBV invades cells and Gn/Gc proteins are more accessible to antibodies.

## Genotyping of DBV by different methods and the comparison

### Classifying strains into Chinese and Japanese lineages (C–J) by geographical distribution

Takahashi et al. found that isolates from Japan and China showed a close phylogenetic relationship in the evolutionary tree, but the strains were clustered into two branches, i.e., Chinese and Japanese lineages. This study used complete genome sequences, and phylogenetic trees were constructed using the neighbor-joining method [52].

In another study, Yoshikawa et al. performed a phylogenetic analysis of 140 L segments (87 strains identified in China, 1 strain identified in South Korea, 52 strains identified in Japan), 171 M segments (91 strains identified in China, 28 strains identified in South Korea, 52 strains identified in Japan), and 211 S segments (125 strains identified in China, 3 strains identified in South Korea, and 83 strains identified in Japan). Chinese and Japanese lineages were further divided into sublineages C1–C5 and J1-J3. Most isolates of the Chinese lineage came from China, whereas isolates of the Japanese lineage came from Japan and Korea. In addition, three Japanese isolates were clustered into the Chinese branch, and four Chinese and 26 Korean isolates were clustered into the Japanese branch. Phylogenetic trees used to analyze molecular evolution were constructed using the Maximum Likelihood Method, and complete genome sequences were used [53]. In the study by Lv et al. [54] two novel sublineages were identified: C6 and J4. Phylogenetic analysis of the L, M, and S gene segments of the complete genome sequences was performed using RAxML. Notably, different genotyping methods have resulted in different numbers and types of genotypes for the S, M, and L segments. Eight sublineages (C1–C4 and J1–J4) have been reported for the S segment, eight sublineages (C1–C5 and J1–J3) for the M segment, and nine sublineages (C1–C6 and J1–J3) for the L segment. Sublineage J4 has only been identified for the S segment, but sublineages C5 and C6 have not been identified for the S segment. Sublineage C6 has only been identified for the L segment.

The source of the strain can be displayed intuitively by naming it by region, such as the C-J genotyping method. However, as DBV gene exchange becomes more frequent and more variations arise, new genotypes may become less region-associated. As a result, genotyping based on geographical distribution may lead to misinterpretation.

### Classifying strains into six genotypes (A–F) by phylogenetic analysis

In the study by Fu et al., 17 strains were isolated from patients with SFTS who lived in Zhoushan, Zhejiang, and whole-genome sequencing was performed for all 17 strains. These 17 strains and another 188 strains from GenBank were included in the phylogenetic analysis. Neighbor-joining, maximum parsimony, and maximum likelihood were considered, and trees were reconstructed. The results revealed that these isolates were attributed to six genotypes (A–F). The mean genetic distance within the genotypes was 0.001–0.026 cM and the inter-genotype mean genetic distance was 0.035–0.062 cM. Genotypes A, D, and F were dominant in China, and a smaller number of isolates belonged to genotype E. Three genotypes (D, E, and F) were present in Korea, and Japan was almost exclusively dominated by genotype B. [46]. Subsequently, Yun et al. reported that genotype B could be further divided into sub-genotypes B1, B2, and B3. In this study, phylogenetic analyses were conducted by aligning published full-length sequences and using the Maximum Likelihood Method, based on the Kimura two-parameter model [10]. The A–F genotyping method is currently the most widely used method for DBV genotyping.

### Classifying strains into five genotypes (A–E) by phylogenetic analysis

Liu constructed an evolutionary tree based on 122 strains using MEGA v5.02 software with the Maximum Likelihood Method. All the sequences used in this study were full-length. Phylogenetic analysis demonstrated that all segments could be divided into five lineages (A, B, C, D, and E). DBV strains from China were distributed among the five genotypes. The Korean DBV strains belonged to genotypes A, D, and E, while the Japanese DBV strains belonged to only genotype E. A total of 55 viral strains belonged to genotype A, among which 54 strains came from China (Henan, Jiangsu, Shandong, Anhui, and Liaoning provinces), while only one strain was isolated in South Korea. All genotype B strains were isolated in China (Henan, Jiangsu, Shan East, Anhui, and Liaoning provinces). Genotype C contained only two strains isolated from Shandong and Jiangsu provinces. Most of the genotype D strains came from China (Henan, Hubei, Shandong, Anhui, and Liaoning provinces), and only one strain came from South Korea. Strains of genotype E came from Japan, the Zhoushan Islands, and South Korea [42]. The B and D genotypes in the study by Li et al. corresponded to the D and B genotype in the study by Liu et al., respectively. In the present study, we conducted our analysis in accordance with Liu et al. This study also used whole-genome sequences of DBV strains. Phylogenetic trees were generated using the Maximum Likelihood Method from the MEGA software [55].

The number of genotypes A–E was lower than that found in studies using the other two genotyping methods. Moreover, it did not report subtypes, but studies using other methods did. The two genotyping methods A–E and A–F employed many identical capital letters, but some identical letters represented different genotypes, thus making it difficult to distinguish the results based on the capital letters used.

### Comparison of different DBV genotyping methods

Owing to time constraints and different sources of the viral isolates, each study used different datasets. These differences resulted in different standards and nomenclatures for DBV genotyping. Further, since DBV contains three segments, researchers usually uploaded the viral segment sequences independently to databases such as GenBank, making subsequent identification of segment sequences from the same strain challenging. Moreover, the genotyping methods classified each segment independently; thus, segments from the same viral strain may be classified as different genotypes. For example, Yun et al. classified the S (MG737277), M (MG737169), and L (MG737060) segments of strain 16MS299 as subtypes B1, B2, and B1, respectively [10]. In addition, reassortment and recombination were not taken into account in most of these genotype designations.

**Fig. 2:**
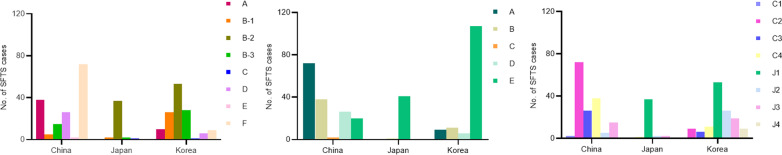
Distribution of DBV genotypes in different countries by different genotyping methods. The dataset used are from Yun et al. [10]. Cases were reported from China, Japan and Korea. Graphs are shown in the order of genotyping methods A–F, A–E and C–J

The correspondence among results by three genotyping methods are shown in Table 2 [10, 42, 54, 55]. The numbers of strains were counted according to geographical distribution and different genotypes (Fig. 2).

**Table 2:** The correspondence among results by three genotyping methods

S			M			L		
Yun et al. (A–F)	Liu et al. (A–E)	Lv et al. (C–J)	Yun et al. (A–F)	Liu et al. (A–E)	Lv et al. (C–J)	Yun et al. (A–F)	Liu et al. (A–E)	Lv et al. (C–J)
A (48)	B (48)	C4 (48)	A (46)	B (46)	C4 (47)	A (46)	B (46)	C4 (46)
B-1 (32)	E (32)	J2 (32)	B-1 (22)	E (22)	J2 (22)	B-1 (22)	E (22)	J2 (22)
B-2 (90)	E (90)	J1 (90)	B-2 (92)	E (92)	J1 (92)	B-2 (86)	E (86)	J1 (86)
B-3 (47)	E (47)	J3 (38)/J4 (9)	B-3 (55)	E (55)	J3 (55)	B-3 (55)	E (55)	J3 (35)
C (2)	B (2)	C4 (2)	C (1)	B (1)	C5 (1)	C (3)	B (3)	C5 (3)
D (32)	D (32)	C3 (32)	D (27)	D (27)	C3 (26) C5 (1)	D (32)	D (32)	C3 (32)
E (2)	C (2)	C1 (2)	E (2)	C (2)	C1 (2)	E (2)	C (2)	C1 (2)
F (81)	A (81)	C2 (81)	F (87)	A (87)	C2 (87)	F (82)	A (82)	C2 (82)
			Unassigned (1)	E (1)	*	Unassigned (7)	E (2)	C6(5)

The data in parentheses are cited from Yun et al. [10]. These numbers denote the number of cases in every genotype.* means that there is no correspondence

As shown in Table 2, subtypes of genotypes B (including B1 and B3) and subtypes of lineages C (including C5 and C6) and J (including J3 and J4) corresponded with different segments. In the genotyping method that classified DBV into genotypes A–F, subtype B-3 (A–F) corresponded to sublineages J3 and J4 (C–J) for the S segment, whereas subtype B-3 (A–F) only corresponded to sublineage J3 (C–J) for the M and L segments. This was due to the absence of sublineage J4 for the M and L segments. Genotype C (A–F) corresponded to sublineage C4 (C–J) for the S segment, but to sublineage C5 (C–J) for the M and L segments.

In the genotyping method that classified DBV into genotypes A–E, genotype B (A–E) corresponded to sublineage C4 (C–J) for the S and L segments, but corresponded to sublineage C5 (C–J) for the M segment. Genotype E (A–E) corresponded to sublineages J1, J2, J3, and J4 (C–J) for the S segment, but to sublineages J1, J2, and J3 (C–J) for the M segment, and to sublineages C5, C6, J1, J2, and J3 (C–J) for the L segment.

When analyzing phylogenies using different sequence datasets, the phylogenetic trees will differ because of differences in the sequence datasets, including the location of certain sequences in the evolutionary tree and the number of sequences corresponding to each genotype.

These genotyping methods relied on phylogenetic tree analyses without specific criteria. The results of these methods did not align well. Determining the correspondence between various genotypes is necessary when referring to multiple studies. This situation must be improved to facilitate research on the molecular biology and immunology of DBV. Establishing a unified, reliable, and practical genotyping method for DBV will largely solve this problem.

## Distribution of DBV genotypes

**Fig. 3:**
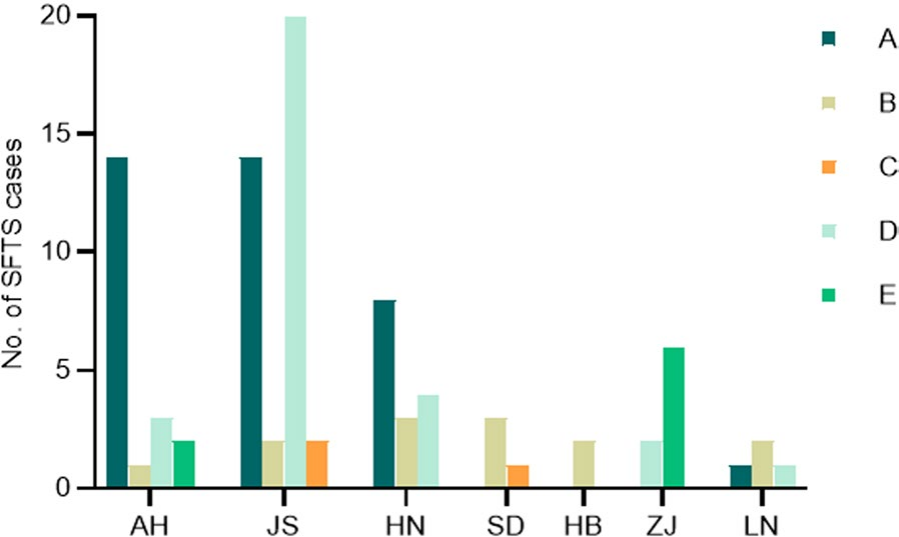
Distribution of DBV genotypes in different regions of China. Data for Fig. 3 is cited from Li et al. [55]. The genotyping method is A–E. The abscissa denotes provinces in China. *AH* Anhui province; *JS* Jiangsu province; *HN* Henan province; *SD* Shandong province; *HB* Hubei province; *ZJ* Zhejiang province; *LN* Liaoning province

Studies reporting the distribution of DBV in populations show that the most prevalent genotype in Japan and Korea is genosubtype B-2 (genotyping method by Fu et al., A–F method), accounting for 86% of cases in Japan and 36.1% of cases in Korea. Genotypes D and E are also distributed in Korea, whereas genotype B is distributed only in Japan. Genotype F is the most common genotype in China, accounting for 44.1% of all cases, followed by genotype A, which accounts for 19.6% of cases. All genotypes are prevalent in China [7, 42]. The numbers of cases per DBV genotype in different regions are shown in Figs. 2 and 3. One of the phylogenetic analyses reported that 83.6% of DBV cases in Jiangsu and Anhui provinces belonged to genotypes A and D (genotyping method by Liu et al., A–E method) and a few cases belonged to genotype E (three cases) [55]. In 2018 and 2020, studies on ticks in Korea showed that the most dominant genotype of DBV was genotype B (genotyping method by Fu et al., A–F method) [56, 57]. In 2019, a study investigated the genotypes of S segments in infected wild pigs in Korea. The study found 33 S segments belonging to genosubtype B-3 and seven S segments belonging to genotype D in DBV-positive serum samples (genotyping method by Fu et al., A–F method) [58].

Strain JaOF2017-7 was discovered in southern Osaka, Japan, in 2017. Phylogenetic analysis showed that segments L and M belonged to sublineage C5, but the S segment belonged to sublineage C4 (genotyping method by Yoshikawa et al., C–J method) [59]. Sublineage C5 belongs to the Chinese branch, which has scarcely been reported in Japan. Some birds can carry DBV-infected ticks, leading to long-distance transmission; thus, sublineage C5 may have been transmitted from China to Japan via birds [60]. In Korea, a study of deer carcasses revealed a close relationship between deer- and human-derived strains, and the M segment of three positive cases was determined to belong to genotype B (genotyping method by Fu et al., A–F method), which is the dominant genotype in Korea. However, the S segment of sample 17WD068 was classified as genotype A, which is mainly prevalent in China. This result shows that the two genotypes may co-circulate in China and Korea [61]. DBV hosts are widely distributed in the wild, and wild animals and their local populations share the same genotype distribution. Therefore, DBV-infected wild animals may remain in a state of occult infection. This long-term carrier state may provide more opportunities for recombination and reassortment among different genotypes. Thus, infected ticks and wild animals can serve as ideal mediators for recombination and reassortment. Surveillance of DBV infection in wild animals can help to better understand and monitor the evolution of DBV.

In the studies on wildlife mentioned above, some were based on partial genomic sequence data. This may have caused a certain bias in the results, such as missing the occurrence of genome reassortment and important point mutation information; however, the partial genomic sequence contained some open reading frames with important mutation sites. In light of economic factors and medical technology levels, partial sequencing results are valuable. To provide a reference for follow-up research, we included them in the review.

The genotypes of DBV are closely correlated with geographic region. The presence of a foreign genotype should be considered when identifying the strain from another endemic zone, as human cases of SFTS are characteristic of an acute disease course, and thus, the probability of interregional transmission of DBV by human carriers is low[62, 63]. Therefore, attention must be paid to the surveillance of virus-harboring wild animals, which may be influenced by factors such as population flow, trade of live animals, and animal migration, thus potentially affecting the distribution of genotypes in countries or regions. In fact, information regarding the distribution of genotypes has provided support in epidemiological studies, leading to a better understanding of the geographical spread of DBV [42, 64].

## Reassortment and DBV genotyping

Reassortment is an important mechanism through which RNA viruses generate new genomes. Reassortment maintains ORF integrity. Thus, the amino acid sequence remains unchanged but the entire genome segment is exchanged [65]. Reassortment is ubiquitous in segmented RNA viruses and can generate new genotypes from existing genotypes. These variants can be more competitive than previous strains, just as some influenza A viruses circulating in pigs or birds have reassorted and gained the ability to infect humans. This example illustrates that reassortment events can result in new genotypes that are capable of cross-species transmission. In addition, reassortment can also render viruses resistant to antiviral drugs and cause pandemics and/or higher fatality rates in the population [66, 67].

When the L, M, and S segments of the same strain belong to different genotypes, it is assumed that the strain has undergone reassortment [68]. Fu et al. identified seven types of reassortment from 10 isolates of DBV from mainland China [46]. Liu et al. [42] found 14 reassortment events in 122 strains spanning 2010–2014. Lv et al. [54] identified 26 possible reassortment events that could be classified into 12 different reassortment types. Furthermore, reassortment between genotypes A and D has been identified [55]. Thus, reassortment in DBV has been shown to be widespread and can lead to novel combinations of segments. We can track the ancestors of DBV and their transmission routes by analyzing the geographical distribution of genotypes and information about strain reassortments. Reassortment plays an important role in the evolution of DBV since the biological characteristics of DBV can be affected by combining segments from different genotypes. However, very few experimental studies on the pathogenicity and virulence of different genotypes of DBV have been conducted, and the specific impacts caused by reassortment need to be explored.

We believe that the main reason for the reassortment of DBV is its wide host range. Therefore, natural selection pressure is also relevant [69]. Segmented RNA viruses, such as the influenza virus, are highly susceptible to reassortment. This is of great significance for both evolutionary and adaptive survival. The migration of the virus may promote the exchange of genes between different locations, thus promoting the emergence of new genotypes. In view of the fact that DBV can be migrated in multiple ways through different hosts, it is also one of the factors that leads to the high incidence of reassortment. Additionally, sequencing errors caused by sample processing and instrument differences are inevitable. However, research on the evolution of viruses is based on large-sample analysis. When selecting the whole genome sequence for genotype analysis, we can preliminarily judge the sequencing quality by reading the literature and retrieving detailed information from the sequencing data.

## Recombination and DBV genotyping

In RNA viruses, recombination is an important mechanism for generating new genotypes [70]. Recombination usually leads to changes in ORFs and gene products and may be identified at any location within RNA virus genomes [71]. The recombination rate of negative-strand segmented RNA viruses is lower than that of positive-strand RNA viruses [72]. A few cases of homologous recombination have been reported in some viruses, such as the influenza A virus, but the role of homologous recombination in virus evolution is limited [73, 74].

However, hantavirus, a member of the order *Bunyavirales*, can undergo homologous recombination [75], which has also been confirmed during the evolution of DBV. Lam et al. [68] discovered chimeric genotypes in the L segment, which was the first confirmation that homologous recombination has played a role in the evolution of DBV. He et al. [76] identified recombination events in the M segment of the DBV. Lv et al. [54] identified 20 recombination events in the genomes of 14 DBV strains, discovering a recombination event in the S segment for the first time. These lines of evidence may prove that homologous recombination leads to the generation of DBV subtypes.

Obviously, homologous recombination may gradually increase the genetic diversity of DBV and cause changes in its antigenicity and virulence; however, the exact mechanisms and impacts of homologous recombination need to be investigated.

## Genotypes and clinical characteristics

### Clinical manifestations and lethality of DBV infection in the population

The clinical symptoms of patients with SFTS may be related to infection with different DBV genotypes. The mortality rates of SFTS in Japan and South Korea are 35% and 23.3%, respectively [77]. In contrast, the mortality rate in China was found to be as low as 6.18%. The most prevalent genotype in Japan and Korea is subtype B-2 (genotyping method by Fu et al.), accounting for approximately 86% and 36.1% of all infections, respectively. This suggests that different genotypes have different fatality rates among the populations. Yun et al. summarized the mortality rates of different genotypes in a population. The mortality rate of subtype B-2 (43.8%, 21/48) was significantly higher than that of the other genotypes, whereas that of genotype A was lowest (10%, 1/10). The mortality rate of genotype F was 44.4% (4/9); however, the number of patients was small. Subtype B-3 had a mortality rate of 28.6% (8/28) and the mortality rate of subtype B-1 was 18.8% (3/16) [10].

A recent study explored the differences in clinical manifestations and laboratory findings in patients from Henan and Zhejiang provinces. The DBV strains causing infections in Henan province mostly belonged to genotypes A, B, and D (genotyping method by Liu et al., A–E method) [42]. Most patients from the Zhoushan Islands in Zhejiang province were infected with genotype E. Higher blood viral load was detected in Henan patients than in Zhejiang patients. In the non-survival group, patients from Henan presented a higher frequency of fever, chills, headache, asthenia, muscle soreness, appetite loss, and lymphadenopathy than patients from Zhejiang. In patients from Henan with SFTS, the dynamic curve of creatinine levels was N-shaped. However, the creatinine levels patients from Zhejiang first showed a slow decline and then a rapid rise. The pattern of rapid increase followed by a slow decrease may be related to the biological characteristics of genotype E. The creatinine level is an indicator of kidney damage. This suggests that genotype E of DBV may be more prone to attack kidney cells and cause more severe organ damage. But we assumed this phenomenon may be caused by kidney injury secondary to systemic disease such as hypotension, shock, DIC, etc. The dynamic curve of alanine aminotransferase (ALT) and aspartate aminotransferase (AST) levels in patients from Henan with SFTS was N-shaped (rising, falling, and then rising until final death). However, ALT and AST levels in the non-survival group of patients from Zhejiang showed a bimodal trend. High ALT and AST levels may reflect hepatic injury. These data suggest a close interaction between the genotype of DBV and the human liver [78].

Patients from Henan may have been infected with more than one genotype of DBV, but the existing research, data collection, and analysis are insufficient. However, the current data indicates that the clinical characteristics of patients with SFTS differ in coastal and inland areas of China. The possibility that genetic background, living environment, and treatment methods may contribute to these differences cannot be excluded. Determining whether and how genotype is related to the mechanism by which the virus causes damage and induces symptoms in the host will be interesting. Finally, more clinical data will lend power to our efforts to elucidate the relationships among viral genotypes, clinical presentations, and prognosis.

### Replication capacity of different genotypes

In an attempt to determine the infectious activity of DBV-infected Vero E6 cells, Yun et al. reported that subtype B-1 (genotyping method by Fu et al., A–F method) showed the highest peak titers. The peak titers of genotypes B-2, B-3, R-1 (B1, B2, and B1 in the order of S, M, and L segments, respectfully), R-2 (B2, B2, and B1), R-3 (B1, B3, and B3), R-4 (B3, F, and B3), and R-6 (D, B1, and D) were found to be lower than those of subtype B-2. Genotypes A, D, F, R-5 (C, D, and C), R-7 (B1, B2, and genotype unknown), R-8 (B1, B3, and genotype unknown), and R-9 (B1, genotype unknown, and genotype unknown) showed the lowest titers in infected cells. Thus, in vitro infection experiments have revealed that different DBV genotypes exhibit different replication efficiency [10].

### Different genotypes of DBV might induce different effects in experimental animals

In an DBV infection animal study, infection of aged ferrets with genotype B, including subtypes B1, B2, and B3 (genotyping method by Fu et al., A–F method), and genotype D resulted in the death of all animals within 12 days of infection. High fever and significant weight loss occurred in > 20% of all animals and a high copy number of viral RNA was detected in these ferrets. Mortality was lower in aged ferrets infected with genotypes A (40%, 5/2) and F (60%, 5/3). In the reassortment genotype groups (genotypes are arranged in the order of S, M, and L segments), infection with genotypes R-2 (B2, B2, and B1), R-3 (B1, B3, and B3), and R-5 (C, D, and C) caused 100% mortality and high fever. Higher peak viral RNA copy numbers were found in comparison to other reassortment genotypes. Infection with genotypes R-1 (B1, B2, and B1), R-4 (B3, F, and B3), R-6 (D, B1, and D), and R-7 (B1, B2, and genotype unknown) caused 80% (4/5), 60% (3/5), 40% (2/5), and 40% (2/5) of mortality in aged ferrets, respectively. Infection with genotypes R-8 (B1, B3, and genotype unknown) and R-9 (B1, genotype unknown, and genotype unknown) caused only 20% (5/1) mortality, low fever, and slightly elevated viral RNA copy numbers. Thus, DBV infections with genotypes B and D show higher mortality and more severe clinical manifestations, such as weight loss and fever [10].

Li et al. used two major viral genotypes to infect spotted doves: strain JS2014-16 (genotype B, genotyping method by Fu et al., A–F method) and strain JS2010-14 (genotype F), which are prevalent in East Asia. When infected with 10^3^ PFU, viremia occurred more often in pigeons infected with the Japanese strain JS2014 (5/8) than in those infected with the Chinese strain js2010 (2/8). When infected with 10^7^ PFU, pigeons infected with strain JS2014-16 had a mortality rate of 12.5% at 6.8 days, with an average peak virus titer of 10^6.9^ PFU/mL. All pigeons infected with the JS2010-14 strain survived for more than 6.1 days with an average peak titer of 10^5.6^ PFU/mL, which was significantly lower than that of pigeons infected with strain JS2014-16. Moreover, infection with strain JS2014 caused earlier and more severe weight loss [12].

These results indicated that strain JS2014-16 may be more virulent than strain JS2010-14. It can be inferred from these results that genotype B induces high mortality and has high pathogenicity, which is consistent with the statistics of DBV infection in the human population. This study provided clear evidence supporting differences in virulence among different genotypes.

## Conclusion

DBV has been identified in several countries. In China, the genetic and genotypic diversity of DBV are relatively high. Reassortment and recombination are important driving forces for the evolution of DBV. Reassortment may lead to novel combinations of genome segments derived from different parental strains, and this phenomenon has been found to be common in DBV. Several attempts have been made to genotype DBV. However, the current DBV genotyping system is confusing because of the lack of uniform standards among different genotyping methods. The establishment of a standard genotyping method based on a large and representative sequence database is urgently required for the following reason: Firstly, the RdRp of RNA viruses lacks a proofreading function; as a result, mutations can be easily generated in the genome of RNA viruses. Genetic mutations in DBV have been associated with changes in certain biological functions, such as viral infection and replication. Studies on mutations at the amino acid level will improve our understanding of the functions of individual viral proteins and their mechanisms and roles in viral pathogenesis. Secondly, animal studies have shown that different DBV strains have different symptoms and fatality rates. These observations strongly indicate that different genotypes of DBV might differ in their pathogenicity, but very few studies have examined this issue to date. Accurate genotyping can help elucidate the relationship between different virus strains and their clinical manifestations. Furthermore, previous studies have indicated that the long-distance spread of DBV is associated with migratory bird movements. Researchers can track changes in viral genotypes in the local environment. Thus, genotyping can provide guidance for the identification of new or imported cases. In summary, reliable genotyping will help to lay a solid foundation for the systematic investigation of the epidemiology, biology, and pathogenesis of DBV.

## Data Availability

All data can be found in the cited references which are publicly available.

## Abbreviations

DBV: *Dabie bandavirus*
ICTV: International Committee on Taxonomy of Viruses
SFTS: Severe fever with thrombocytopenia syndrome
SFTSV: Severe fever with thrombocytopenia syndrome virus
HYSV: Huaiyang Shan virus
NBV: New bunyavirus
RdRps: RNA-dependent RNA polymerases
ORF: Open reading frame
GP: Glycoprotein
DC: Dendritic cell
DC-SIGN: Dendritic cell (DC)-specific intracellular adhesion grabbing non-integrin
NSs: Nonstructural protein
NP: Nucleocapsid protein
RNP: Ribonucleoprotein
RVFV: Rift Valley fever phlebovirus
CCHFV: Crimean-Congo hemorrhagic fever virus
ALT: Alanine aminotransferase
AST: Aspartate aminotransferase
